# Cyclooxygenase-2, a Potential Therapeutic Target, Is Regulated by miR-101 in Esophageal Squamous Cell Carcinoma

**DOI:** 10.1371/journal.pone.0140642

**Published:** 2015-11-10

**Authors:** Ying Shao, Peng Li, Sheng-tao Zhu, Ji-ping Yue, Xiao-jun Ji, Zhen He, Dan Ma, Li Wang, Yong-jun Wang, Ye Zong, Yong-dong Wu, Shu-tian Zhang

**Affiliations:** 1 Department of Gastroenterology, Beijing Friendship Hospital, Capital Medical University, National Clinical Research Center for Digestive Disease, Beijing Key Laboratory for Precancerous Lesion of Digestive Diseases, Beijing, China; 2 Intensive Care Unit, Beijing Friendship Hospital, Capital Medical University, Beijing, China; Medical College of Soochow University, CHINA

## Abstract

**Background & Aims:**

Cyclooxygenase-2 (COX-2) is known to promote the carcinogenesis of esophageal squamous cell carcinoma (ESCC). There are no reports on whether microRNAs (miRNAs) regulate COX-2 expression in ESCC. This study investigated the effect of miR-101 on ESCC through modulating COX-2 expression in ESCC.

**Methods:**

Real-time quantitative reverse transcription–polymerase chain reaction (RT-PCR) was used to quantify miR-101 expression in ESCC clinical tissues and cell lines. The effects of miR-101 on ESCC progression were evaluated by cell counting kit-8 (CCK8), transwell migration and invasion assays, as well as by flow cytometry. The COX-2 and PEG2 levels were determined by western blot and enzyme-linked immunosorbent assays (ELISA). The luciferase reporter assay was used to verify COX-2 as a direct target of miR-101. The anti-tumor activity of miR-101 *in vivo* was investigated in a xenograft nude mouse model of ESCC.

**Results:**

Downregulation of miR-101 was confirmed through comparison of 30 pairs of ESCC tumor and adjacent normal tissues (*P* < 0.001), as well as in 11 ESCC cell lines and a human immortalized esophageal cell line (*P* < 0.001). Transfection of miR-101 in ESCC cell lines significantly suppressed cell proliferation, migration, and invasion (all *P* < 0.001). The antitumor effect of miR-101 was verified in a xenograft model. Furthermore, COX-2 was shown to be a target of miR-101.

**Conclusions:**

Overexpression of miR-101 in ESCC inhibits proliferation and metastasis. Therefore, the miR-101/COX-2 pathway might be a therapeutic target in ESCC.

## Introduction

Esophageal cancer, one of the most common malignant tumors, is the eighth most common cancer and the sixth most common causes of cancer mortality in the world. Histologically, esophageal cancer can be divided into two main forms: esophageal squamous cell carcinoma (ESCC) and adenocarcinoma. Adenocarcinoma is common in western countries but ESCC is predominant in East Asia, especially in China [1]. In China, approximately 210,000 patients die each year of ESCC, or 52% of all ESCC deaths worldwide [2]. Although advances have been made in the treatment of ESCC, the prognosis of ESCC patients remains very poor, and the overall 5-year survival rate of patients with ESCC is still less than 10–15% [3]. Therefore, it is urgent to discover more biomarkers and therapeutic targets for ESCC.

Cyclooxygenases 1 and 2 (COX-1 and COX-2) are the rate-limiting enzymes involved in the biosynthesis of prostaglandins (PGs). COX-1 is constitutively expressed in most tissues, while COX-2 is the inducible isoform, which is responsible for the elevated production of PGs in response to various inflammatory stimuli, hormones, and growth factors [4]. Accumulating evidence has demonstrated that COX-2 plays an important role both in tumor development and progression [5–10], including ESCC [11–16]. Epidemiological studies have indicated that the regular use of aspirin can reduce the risk of esophageal cancer by up to 90% [17–19]. We and others have shown that (1) COX-2 expression is a frequent phenomenon in human ESCC tissue samples and that positive expression is related to lymphatic metastasis [11–16]; (2) COX-2 inhibitors inhibit cell proliferation and induce apoptosis by inducing G0 / G1 cell-cycle arrest and down-regulating Bcl-2 expression [20, 21] and the inhibition of COX-2 leads to tumor reduction *in vivo* [22]; and (3) a COX-2 inhibitor can inhibit migration and invasion of ESCC cells [23] These findings thus provide compelling evidence that COX-2 is an obligatory player in ESCC and that blocking COX-2 is an important therapeutic targets of ESCC.

So far, there are three main COX-2 block methods: COX-2 inhibitors, inhibitory transcription factors and post-transcriptional control. The application of the first two methods is restricted because of the adverse reaction of COX-2 inhibitors [24, 25] and the non-specificity of transcription factors. Because post-transcriptional control has better effect, it is currently the focus of much research. MicroRNAs (miRNAs), which encode small non-coding RNAs of approximately 22 nucleotides, are now recognized as an effective method of post-transcriptional control. Previously, we searched seven databases (Targetscan, Pictar, MiRanda, MiRwalk, Dianamt, Ebi and Microrna) and found four putative miRNAs that could bind to the 3 ‘- untranslated region (UTR) of COX-2 (according to at least five databases), which are miR-101, miR-144, miR-26a and miR-143. Among four these miRNAs, miR-101 has been reported to be down-regulated in an ESCC cell line [26]. Thus, we hypothesized that miR-101 could inhibit ESCC through inhibiting COX-2.

In this study, we validated the down-regulation of miR-101 in ESCC tissue specimens and cell lines; investigated the inhibitory effect of miR-101 on ESCC cell proliferation, migration and invasion *in vitro*; further examined the anti-tumor activity of miR-101 *in vivo* in a xenograft nude mouse model of ESCC; and verified that miR-101 inhibited ESCC via inhibiting COX-2 expression. To the best of our knowledge, this is the first study to examine the miR-101 / COX-2 pathway in ESCC.

## Materials and Methods

### Ethics Statement

The Clinical Research Ethics Committee of Beijing Friendship Hospital, Capital Medical University approved the project and protocol for the investigations involving humans and animals. This study was performed in accordance with the ethical standards of the Declaration of Helsinki. All participants provided their written informed consent to participate in this study, and the ethics committee approved this consent procedure.

### Collection of ESCC clinical samples and culture of ESCC cell lines

Thirty pairs of primary ESCC tissues and the corresponding adjacent normal esophageal tissues were obtained from untreated patients in Beijing Friendship Hospital (Capital Medical University) from 2009 to 2011 with informed consent and agreement. All tissue samples were snap frozen in liquid nitrogen and stored at –80°C until the extraction of RNA.

The human ESCC cell lines KYSE30, KYSE70, KYSE150, KYSE410, KYSE450, KYSE510, EC9706, and EC109 were kindly provided by the Cancer Institute and Hospital, Chinese Academy of Medical Science. The ESCC cell lines TE-1, TE-9, and TE-11 were gifts from Hebei Cancer Hospital of China. All of the ESCC cell lines used in this study was from the Cell Bank of Type Culture Collection of the Chinese Academy of Sciences, Shanghai Institute of Cell Biology and has been described previously [27–31]. Het-1A, a human esophageal immortalized cell line, was purchased from the American Type Culture Collection. Five-week-old male BALB/c nu/nu mice were purchased from the Institute of Laboratory Animal Sciences, Chinese Academy of Medical Sciences. All cell lines, except for Het-1A, were cultured in RPMI-1640 medium (Hyclone, USA) containing 10% fetal bovine serum (Gibco, USA) and 10 units / mL penicillin and streptomycin (Hyclone, USA). Cells were maintained at 37°C, 95% humidity, and 5% CO_2_. Het-1A cells were cultured in bronchial epithelial basal medium with growth supplements (Clonetics, USA).

### Real-time quantitative reverse-transcription polymerase chain reaction (RT-PCR)

To measure the expression levels of mature miR-101, real-time quantitative RT-PCR was performed as described previously [32]. The miRNAs were extracted from cultured cells or frozen tissues with a mirVana miRNA Isolation Kit (Applied Biosystems, USA). Using an Applied Biosystems 7500 Fast System and standard TaqMan PCR reagents, ten nanograms of the extracted miRNAs were reverse-transcribed to cDNA using a TaqMan MicroRNA Reverse Transcription Kit (Applied Biosystems, USA). The reaction mixture was used for real-time RT-PCR of miR-101. U6 was used as an internal control. Fold changes for the expression levels of miR-101 were calculated using the comparative cycle threshold method (2^-ΔΔCT^). All miRNA samples were assayed in triplicate.

### Construction of expression plasmids and stable over-expression clones

The specific primers for establishing expression plasmids of miR-101 were as follows: miR-101 sense: 5 ‘-CG G / GATCC (*Bam*HI) TTCAGCCTCACCACTTGCTG-3 ‘; anti-sense: 5 ‘- CAACATGGCTGCACCAACAAC A / AGCTT (*Hind*III) GGG– 3 ‘ (the restriction sites are underlined in each primer). Precursors of miR-101 (440 bp) were cloned into the pSilencer 4.1-CMV vector (Ambion, Geneworks), in accordance with the manufacturer’s instructions. In this study, we selected EC9706 and EC109 cell lines as our model, since high COX-2 levels in those cells have been found and miR-101 has been reported to be down-regulated in EC109 cells [26]. Introduction of the expression plasmids carrying miR-101 into EC9706 and EC109 cells was performed using Lipofectamine 2000 (Invitrogen) in accordance with the manufacturer’s instructions. Stably expressed cell lines were selected with G418 (Sigma, USA) at a dose of 600 mg / mL and maintained at 300 mg / mL. The expression levels of miR-101 in stable clones were verified via real-time RT-PCR.

### Colony formation assay

Cells were plated in 6-well plates at 1×10^3^ cells / well and grown for 2 weeks. After 2 weeks, the cells were washed twice with phosphate-buffered saline, fixed with methanol, and stained with Giemsa. The number of colonies was counted under the micro-scope.

### Cell proliferation assay

Cell proliferation was measured with a Cell Counting Kit-8 (CCK-8, Dojindo, Japan). A colorimetric assay was used to create growth curves using the mean results from three independent experiments. Briefly, cells were seeded at a density of 5 × 10^3^ cells / well in a 96-well plate. Cell viability was assessed using the CCK8 at 24, 48, 72, 96, and 120 h after transfection. The absorbance at 450 nm was measured with a plate reader.

### Cell cycle analysis and detection of apoptosis

Dissociated cells were fixed and permeabilized with cold 70% ethanol, overnight at 4°C. The next day, the cells were stained with 10 μL of 1 mg / mL propidium iodide and 10 μL of 500 μg / mL RNase (37°C for 30 min) for flow cytometry. In addition, cells stained with Annexin V-FITC and propidium iodide were used to determine the rate of apoptosis by flow cytometry, in accordance with the protocol of the FITC Annexin V Apoptosis Detection Kit I (BD Pharmingen, USA).

### Tumor formation in nude mice

Stably transfected cells (1.5 × 10^6^ in 0.2 mL) were injected subcutaneously into the right (EC9706 / EC109-miR-101) and left (EC9706 / EC109-vector) dorsal flank of severe combined immunodeficiency mice (SCID; Institute of Laboratory Animal Sciences, Chinese Academy of Medical Sciences), five mice per group. At 4 weeks after inoculation of the tumor cells, the tumor size was determined by the formula volume = 0.5 × length × width^2^.

### Cell migration and invasion assays

Cell migration and invasion were analyzed in cells incubated for 24 h using non-Matrigel-coated (BD Falcon cell culture inserts, BD Biosciences, USA) or Matrigel-coated transwell cell culture chambers (BD Matrigel Invasion Chamber, BD Biosciences, USA), of 8 μm pore size, in accordance with the manufacturer’s instruction.

### Metastasis assay in nude mice

Stably transfected cells were injected intravenously (3 × 10^5^ cells / mouse) into 5-week-old SCID mice through the tail vein. There were two groups (EC9706 / EC109-vector and EC9706 / EC109-miR-101) in the animal study and each group had five mice. The number of tumor nodules formed on the surface of the liver, lungs, kidney and spleen was counted at 12 weeks after tumor injection.

### Western blot

Western blot analysis was performed as described previously [33]. The primary antibodies and secondary antibody used in this study were rabbit anti-COX-2 antibody (1:1000, Cell Signaling, USA), rabbit anti-β-actin antibody (1:1000, Sigma, USA), and sheep anti-rabbit antibody conjugated to horseradish peroxidase (1:6000, Santa Cruz, USA).

### Enzyme immunoassay for prostaglandin E2 (PGE2)

PGE2 in the culture supernatant was measured by an enzyme-linked immunosorbent assay (ELISA), in accordance with the kit manufacturer’s protocol. The concentration of PGE2 was expressed as picograms per milliliter.

### Dual-luciferase reporter assay

Luciferase reporters of COX-2 were made through RT-PCR using the following primers: wild type (WT)-binding sites of miR-101 (490 bp), upstream primer with a *Sac*I site (5 ‘- GCCACAGAGCT / C AGCTATCTGTAACCAAGATGGATGC-3 ‘) and downstream primer with a *Hind*III site (3 ‘- GTCCTTAGGATAGGCCTATGTGCTA A / A GCTTCCGCAT- 5 ‘); Luciferase reporters of COX-2 with mutant type (MT)-binding sites (underlined and italicized) of miR-101 were made using fragments with 5 ‘ *Sac*I and 3 ‘ *Hind*III sites (underlined) and protective bases, shown below: GCCACA GAGCT / C AATAATAATGACGATAATACTTCTTTTCCACATCTCATTGTCACTGACATTTAATG ***A*****TA*****A*****T*****A***TATATTACTTAATTTATTGAAGATTATTATTTATGTCTTATTAGGACACTATGGTTATA / AGCTTCCGCAT. PCR products or synthetic DNA fragments were digested and cloned into *Sac*I and *Hind*III sites on a pMIR-REPORT miRNA Expression Reporter Vector (Ambion, USA), which was confirmed by DNA sequencing.

To perform the luciferase reporter assay, EC9706-miR-101 cells (4 × 10^4^) were seeded in a 48-well plate and then co-transfected with 800 ng of either the WT (pMIR-miR-101 WT) or MT(pMIR-miR-101 MT) report or 40 ng of pMIR-vector and 4 ng of pRL-TK vector (Promega, USA), an internal control using Lipofectamine 2000 (Invitrogen). Forty-eight hours after transfection, cells were collected for analysis using a Dual-Luciferase Reporter Assay Kit (Promega, USA), in accordance with the manufacturer’s instructions. The results were measured with a GloMax-Multi Detection System (Promega, USA). Transfection was done in duplicates and experiments were repeated three times.

### Statistical analysis

SPSS 15.0 software (Chicago, IL, USA) was used for statistical analysis in this study. Data were presented as means ± standard deviation (SD), which were collected from at least three independent experiments. The two-tailed Student’s t-test was used for comparisons of two independent groups. *P* < 0.05 was considered statistically significant.

## Results

**Fig 1:**
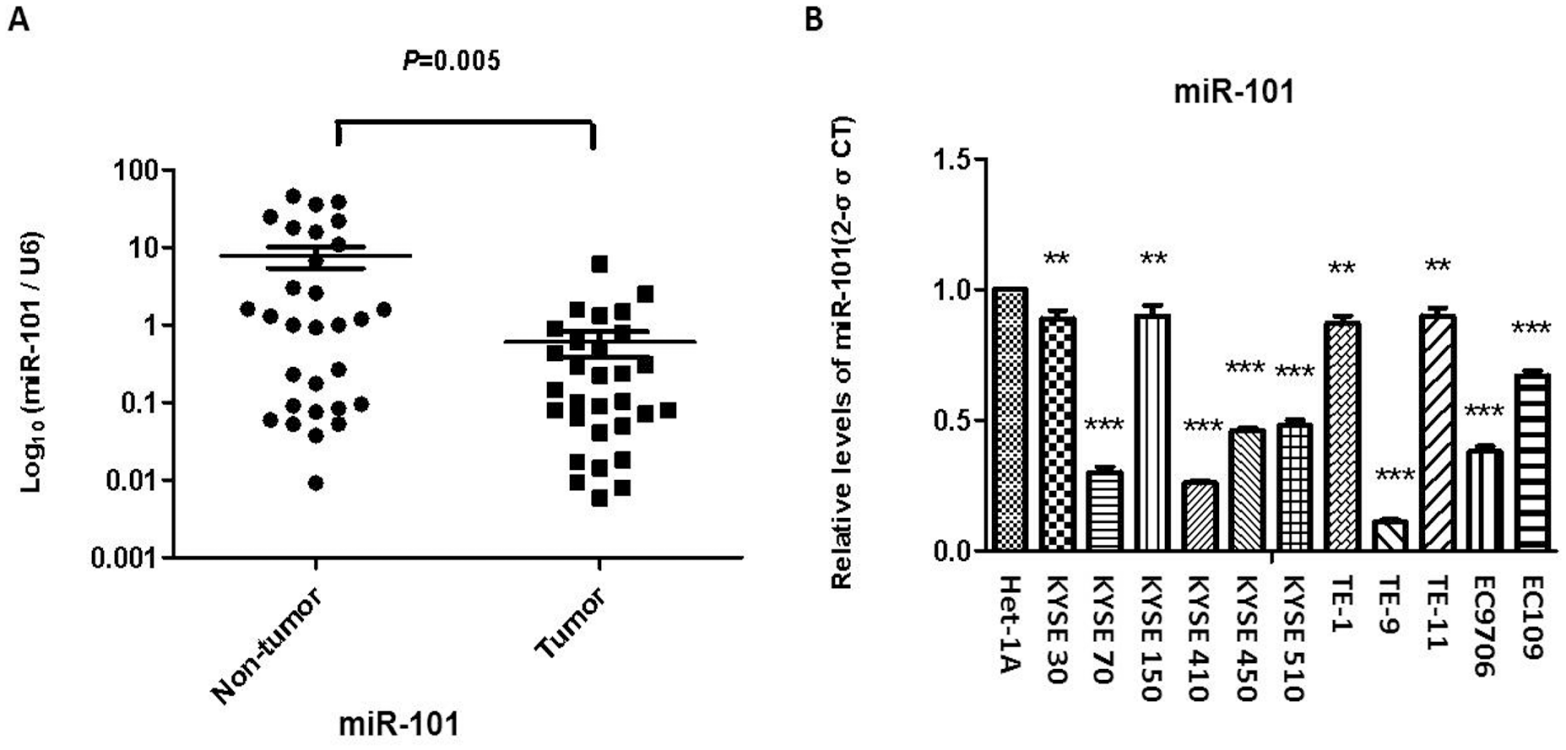
Downregulation of miR-101 in tumor tissue specimens and cell lines of human ESCC. The expressions of miR-101 in 30 pairs of ESCC tumor tissues and corresponding normal tissues (A) and cell lines (B) were determined by quantitative real time RT-PCR as described in Materials and Methods. Results were calculated by 2^-ΔΔCT^ method and shown as the mean value of three independent experiments. U6 was used as an internal control for data normalization of RT-PCR. Columns and error bars represent standard deviations from three independent measurements. ** *P* < 0.01; *** *P* < 0.001.

**Fig 2:**
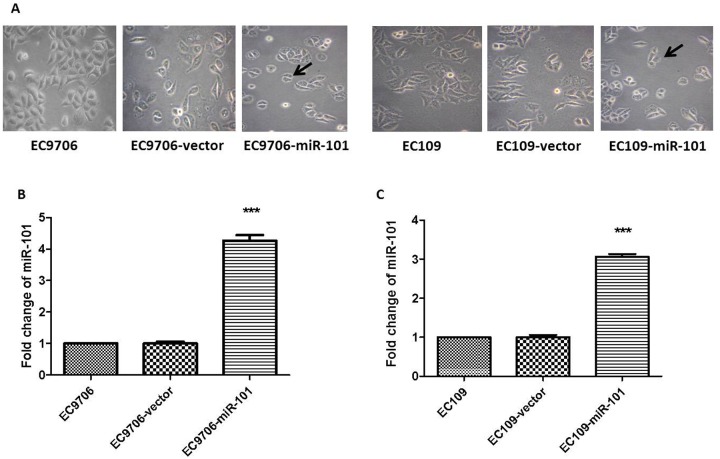
Effects of ectopically expressed miR-101 on the morphology and the expression level of miR-101 of EC9706 and EC109. (A) EC9706 / EC109-miR-101 had change in volume (decreased) compared to the parent cells and cells expressing the vector-control under inverted microscope (40×). Relative expression of miR-101was detected by quantitative PCR in stably transfected EC9706-miR-101 (B) and EC109-miR-101 (C) relative to the parent cells and vectors-control cells. Expression was normalized against an endogenous control U6 (average of two individual experiments with triplicated Samples). *** *P* < 0.001.

**Fig 3:**
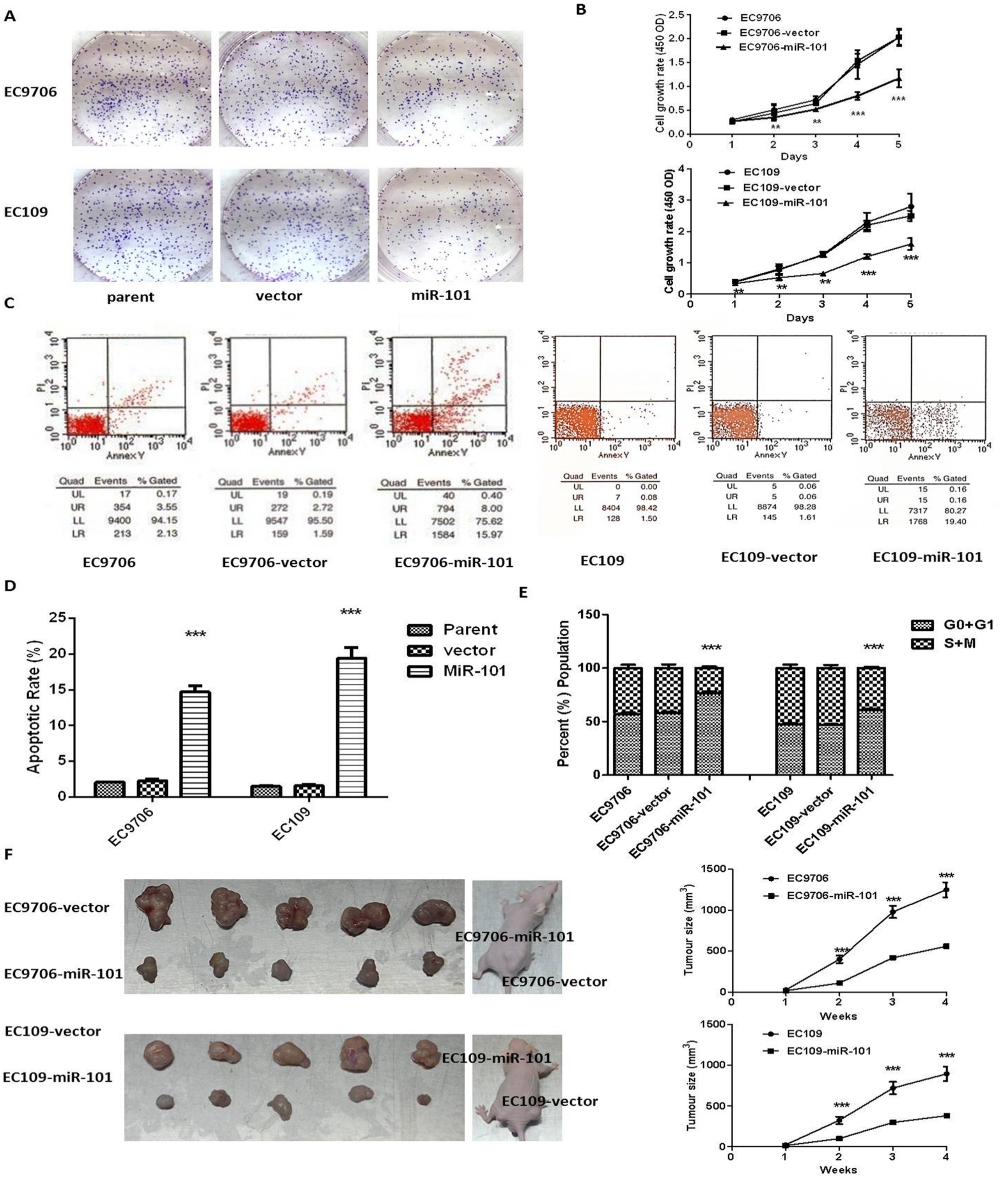
Overexpression of miR-101 inhibited the proliferation of ESCC cell lines in vitro and in vivo. (A) Representative results of colony formation of parent (column 1), vector (column 2), and miR-101–transfected (column 3) EC9706 and EC109. (B) Cell growth curves of EC9706 and EC109 stably transfected with miR-101 were determined by CCK-8 assay. Growth curve was generated by reading the absorbance value at different time points (upper for EC9706 and lower for EC109 cells). (C and D) After 24 hrs culture, medium was replaced by fresh serum-free medium for additional 12 hrs culture. At endpoint, cells were collected and stained with Annexin V-FITC and propidium iodide for flow cytometric assay of apoptosis (left for EC9706; right for EC109). (E) Representative cell cycle analysis of parent, vector and miR-101-transfected EC9706 and EC109 (left for EC9706; right for EC109). (F) 1.5 × 10^6^ cells stably transfected with vector or miR-101 were implanted subcutaneously in the left and right dorsal flank areas of nude mice, respectively, (5 mice per group). Tumor volume was measured once a week over a period of 4 weeks. The results were expressed as mean ± SD of the results of three independent experiments, each with triplicates. ** *P* < 0.01; *** *P* < 0.001.

**Fig 4:**
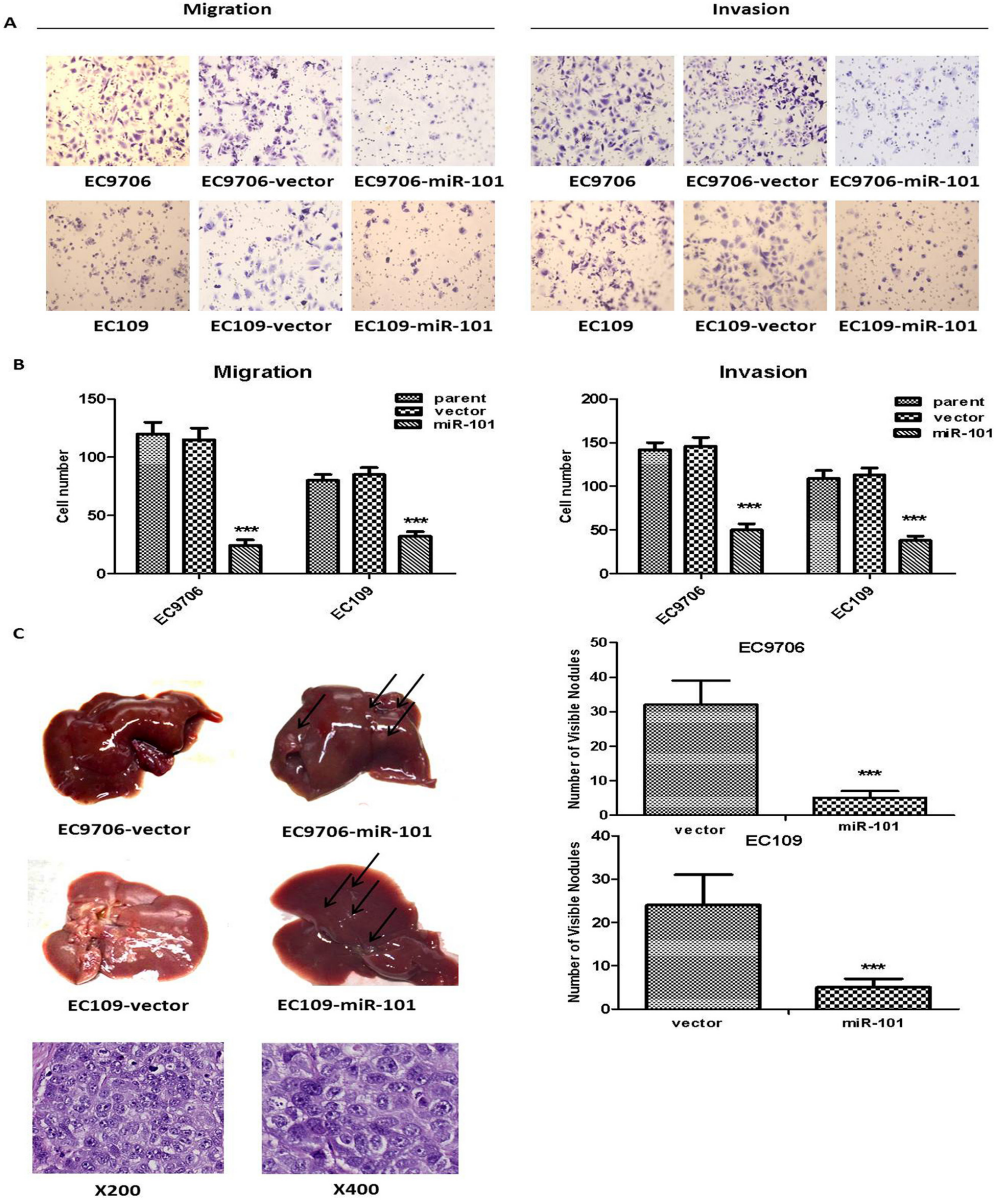
Ectopic expression of miR-101 inhibited the metastasis of ESCC. (A and B) The migration and invasion ability (24 hrs) of tumor cells was determined by transwell chamber assay as described in Materials and Methods (left for migration; right for invasion). (C) 3 × 10^6^ cells were inoculated into nude mice by tail vein injection. Twelve weeks after inoculation, mice were killed and metastatic nodules on the surface of liver were counted. Hematoxylineosin staining showed the nodules on the liver were metastasis. The results are expressed as mean ± standard deviation of data from three independent experiments, each with three repetitions, ***, *P* < 0.001.

**Fig 5:**
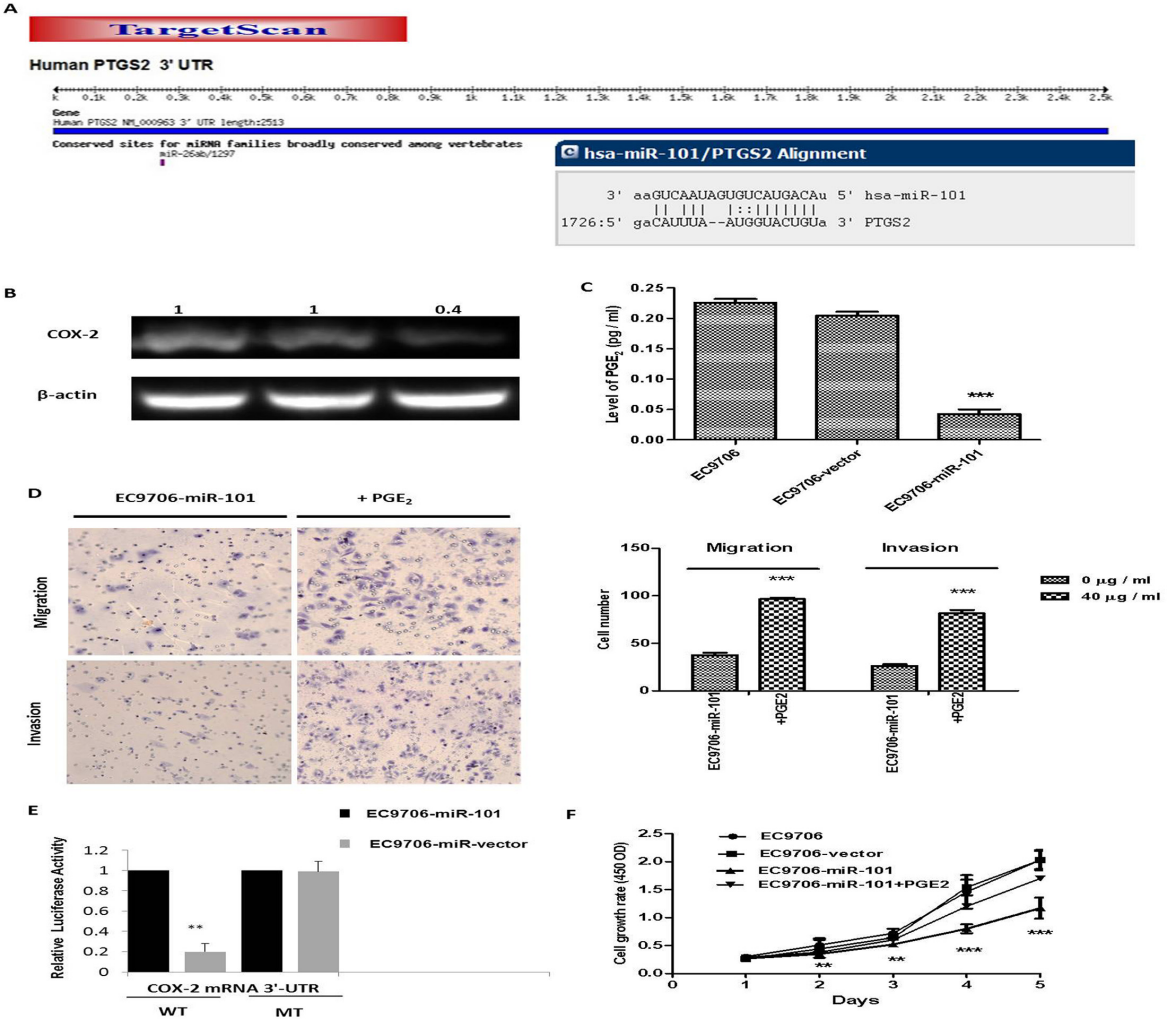
MiR-101 inhibits ESCC via COX-2. (A) The predicted binding sites of hsa-miR-101 in the 3’-UTR of COX-2 mRNA and their mutant counterparts. (B) COX-2 expression in stably transfected cell lines was detected by western blot analysis as described in Materials and Methods. Beta-actin was used as an internal control for COX-2 expression normalization. (C) Culture medium was collected for measurement of PGE2 levels by using ELISA assay as described in Materials and Methods. (D) Similar to protocol used in Figs 3B and 4A, but cells were cultured in serum-free medium supplemented with 40 μg / mL PGE2 for 24-h. The abilities of tumor cell proliferation, invasion and migration in the presence of PGE2 increased significantly. (E) Growth curve of the EC9706- miR-101 cell line that was cultured in the medium supplemented with 40 μg / mL PGE2. (F) After 48h transfection of pMIR-COX-2-WT or pMIR-COX-2-MT, luciferase assay was measured. Renilla luciferase was used for normalization. The data are an average of triplicate samples from three independent experiments. ** *P* < 0.01; *** *P* < 0.001.

1. MiR-101 is down-regulated in ESCC. The expressions levels of miR-101 in clinical specimens of ESCC and the corresponding adjacent normal tissues obtained from 30 patients with ESCC, as well as in 11 ESCC cell lines and a human immortalized esophageal epithelia cell line (Het-1A) were determined. Compared to the adjacent normal tissues and Het-1A cell, the expressions levels of miR-101 were significantly downregulated in tumor tissues (Fig 1A) and 11 ESCC cell lines (Fig 1B).

2. Morphology changes and miR-101 expression levels of in miR-101-transfected ESCC cell lines. To explore the potential tumor-suppressive role of miR-101 in ESCC, the miR-101 precursor was cloned and stably transfected into the ESCC cell lines EC9706 and EC109. First, we observed that both EC9706 and EC109 cells over-expressing ectopic miR-101 exhibited a morphological change with a decrease of volume compared to the typical morphology of parent cells and the vector-control (Fig 2A). Both EC9706 and EC109 cells over-expressing ectopic miR-101 (Fig 2B and 2C).

3. MiR-101 suppresses tumor proliferation *in vitro* and *in vivo*. The colony formation of EC9706-miR-101 and EC109-miR-101 cells was significantly suppressed (Fig 3A). The CCK8 assay results showed that the proliferation rates of the EC9706-miR-101 and EC109-miR-101 cells were significantly less than those of the parent and vector-control cells (Fig 3A and 3B). To investigate the biological function of miR-101 in ESCC, we examined the proliferation of ESCC cells. The CCK8 assay data showed that the proliferation of the EC9706 and EC109 cell lines stably transfected with miR-101 was significantly inhibited (Fig 3B). Flow cytometry revealed that overexpression of miR-101 in the ESCC cell lines significantly induced both apoptosis and cell cycle arrest at the G0 / G1 phase (Fig 3C–3E). In addition, overexpression of miR-101 in the ESCC cell lines significantly inhibited the growth of xenograft tumors in nude mice, as compared with mice inoculated with vector EC9706 and EC109 cells (Fig 3F).

4. MiR-101 suppressed tumor metastasis *in vitro* and *in vivo*. The transwell chamber assay showed that the migration and invasion ability of ESCC cells stably transfected with miR-101 was significantly suppressed, compared with the parent cells or vector-control cells (Fig 4A and 4B). In addition, the number of metastatic nodules on the surfaces of the liver was significantly less in mice inoculated with miR-101-transfected ESCC cells compared to the negative control mice (vector groups) (Fig 4C, *P* < 0.001). No visible metastatic nodules on the lung, kidney, or spleen were found in either the vector group or the miR-101 group.

5. COX-2 is a direct target of miR-101 in ESCC. To verify the regulatory effect of miR-101 on COX-2 expression in ESCC cells, we performed the following experiments. First, the predicted binding sites of hsa-miR-101 in the 3 ‘-UTR of COX2 mRNA were determined according to computational prediction (TargetScan and microRNA databases). Based on this information, luciferase reporters containing mutant binding sites were constructed (Fig 5A), in order to verify whether COX-2 is a direct target of miR-101 in human ESCC. Second, we investigated the correlation of miR-101 with COX-2 expression in EC9706, in which miR-101 was over-expressed in our previous study (data not shown). Western blot analysis showed that overexpression of miR-101 in EC9706 cells significantly reduced COX-2 expression at the protein level (Fig 5B). Furthermore, the level of PGE2, a primary product of COX-2, in stably transfected cells was significantly less than that of parent and vector-transfected cells (Fig 5C; *P* < 0.001). In addition, the proliferation, migration and invasion abilities of miR-101-transfected cells increased with PGE2 supplementation in the culture medium (Fig 5D and 5E; *P* < 0.001). Moreover, the luciferase reporter activity of WT or MT pMIR-COX-2 plasmid in EC9706 cells that overexpressed miR-101 showed that the reporter activity of WT pMIR-COX-2 was significantly decreased in cells that over-expressed miR-101, as compared to the control cells (*P* < 0.01; Fig 5F). The results were expressed as mean ± SD of three independent experiments, each performed in triplicate. ** *P* < 0.01; *** *P* < 0.001.

## Discussion

ESCC is a complex and heterogeneous disease with multiple underlying pathogenic mechanisms caused by various risk factors. Therefore, it is of great significance to identify novel and effective molecular markers for ESCC diagnosis and treatment assessment. In the present study, we investigated the effect and possible mechanism of miR-101 on human ESCC. First, we examined the expression levels of miR-101 in ESCC. A comparison between 30 pairs of ESCC tumor and adjacent normal tissues showed that the expression levels of miR-101 were significantly less in ESCC tumor (*P* < 0.001). Similar results were also found in 11 ESCC cell lines, suggesting that miR-101 is downregulated in human ESCC. Second, we showed that miR-101 can reduce cell proliferation by inducing apoptosis and cell cycle arrest at the G0 / G1 phase, as well as decrease the migration and invasion abilities of ESCC cells. Moreover, we confirmed the inhibitory effect of miR-101 on ESCC using an *in vivo* study. Taken together, all of the data from the *in vitro* and *in vivo* studies indicated that miR-101 inhibited the proliferation and metastasis of ESCC. Thirdly, using the human ESCC cell line EC9706 stably transfected with miR-101, we further validated that COX-2 and PGE2 were downregulated at the protein level by the proposed target miR-101. This finding was also confirmed by a luciferase reporter assay, suggesting that miR-101 directly targets COX-2 in ESCC.

The miRNA profile can reveal prospective features in cancer. In ESCC, dozens of miRNAs have been reported to be involved in tumorigenesis and tumor progression [26, 27, 32, 34–44]. However, the functions and real targets of miRNAs were largely unknown. In the present study, we focused on miR-101, a tumor suppressive miRNA, for three reasons. First, miR-101 is located in the genomic loci with a high frequency of allelic losses in several types of cancers, and the down-regulation of miR-101 has been found in a variety of human malignancies including ESCC [26, 45–61] Second, in our previous study, we found that COX-2 has an important effect on the proliferation and metastasis of ESCC [20–23, 33, 62–63]; in our preliminary experiment, among four putative miRNAs (miR-101, miR-143, miR-26a and miR-144) that could bind to the 3 ‘-UTR of COX-2 predicted by at least five databases (as shown above), only miR-101 could inhibit both the proliferation and metastasis of ESCC. Finally, there are no reports regarding miR-101 targeting COX-2 in ESCC, although the regulation of COX-2 expression by miRNAs has been extensively studied in a variety of human tumors [5, 60, 64–70] and the miR-101 / COX-2 pathway has been reported in colon cancer [70], cervical cancer [57], gastric cancer [60] and prostate cancer [69]. In this study, we found the proliferation, migration, and invasion abilities of EC9706 cells stably transfected with miR-101 increased significantly after the addition of PGE2 (a primary product of COX-2). Moreover, the expression levels of miR-101 and, COX-2 as well as PGE2 were negatively correlated according to the western blot of luciferase reporter assay results, indicating that miR-101 inhibit ESCC through COX-2 and that the miR-101 / COX-2 pathway may be very important in ESCC.

The COX-2 gene is located on chromosome 1q25.2-q25.3, with a length of 8.3 kb, consisting of 10 exons and 9 introns, and encoding 604 amino acid residues. It is now accepted that COX-2 can promote cell proliferation, inhibit apoptosis, promote angiogenesis, and suppress immune function as well as other mechanisms involved in tumor development and progression. Recent progress in suppressor miRNA delivery technology offers the possibility of promising new approaches to block COX-2. Among miRNA-based approaches using an in vivo delivery system with DNA plasmids or viral vectors, miRNA replacement therapy with double-stranded RNAs mimicking miRNAs may be one of the most promising methods. As miR-101 has low-to-null toxicity, it may represent a novel class of COX-2-targeting molecules to be exploited for preventive and therapeutic purposes.

In summary, this study provides new insights into the role of miR-101 in human ESCC. It shows that miR-101 is down-regulated in ESCC tumor tissues and cell lines, and that it is able to inhibit cell proliferation, migration and invasion of ESCC cells *in vitro* as well as reduce xenograft tumor growth and the number of metastatic nodules on the surface of the liver *in vivo* by targeting COX-2. These results suggest that miR-101 is a tumor suppressor in ESCC, and that it might serve as a therapeutic target in ESCC.
